# ‘Remote’ behavioural ecology: do megaherbivores consume vegetation in proportion to its presence in the landscape?

**DOI:** 10.7717/peerj.8622

**Published:** 2020-02-19

**Authors:** Christopher G. Marston, David M. Wilkinson, Matt Sponheimer, Daryl Codron, Jacqui Codron, Hannah J. O’Regan

**Affiliations:** 1 Land Use Group, UK Centre for Ecology and Hydrology, Lancaster, UK; 2 School of Life Sciences, University of Lincoln, Lincoln, UK; 3 Department of Classics and Archaeology, University of Nottingham, Nottingham, UK; 4 Department of Anthropology, University of Colorado at Boulder, Boulder, USA; 5 Department of Zoology and Entomology, University of the Free State, Bloemfontein, South Africa; 6 Centre for Environmental Management, University of the Free State, Bloemfontein, South Africa

**Keywords:** Behavioural ecology, Diet, Elephant, Isotope, Kruger National Park, Landsat, Remote sensing

## Abstract

Examination of the feeding habits of mammalian species such as the African elephant (*Loxodonta africana*) that range over large seasonally dynamic areas is exceptionally challenging using field-based methods alone. Although much is known of their feeding preferences from field studies, conclusions, especially in relation to differing habits in wet and dry seasons, are often contradictory. Here, two remote approaches, stable carbon isotope analysis and remote sensing, were combined to investigate dietary changes in relation to tree and grass abundances to better understand elephant dietary choice in the Kruger National Park, South Africa. A composited pair of Landsat Enhanced Thematic Mapper satellite images characterising flushed and senescent vegetation states, typical of wet and dry seasons respectively, were used to generate land-cover maps focusing on the forest to grassland gradient. Stable carbon isotope analysis of elephant faecal samples identified the proportion of C_3_ (typically browse)/C_4_ (typically grass) in elephant diets in the 1–2 days prior to faecal deposition. The proportion of surrounding C_4_ land-cover was extracted using concentric buffers centred on faecal sample locations, and related to the faecal %C_4_ content. Results indicate that elephants consume C_4_ vegetation in proportion to its availability in the surrounding area during the dry season, but during the rainy season there was less of a relationship between C_4_ intake and availability, as elephants targeted grasses in these periods. This study illustrates the utility of coupling isotope and cost-free remote sensing data to conduct complementary landscape analysis at highly-detailed, biologically meaningful resolutions, offering an improved ability to monitor animal behavioural patterns at broad geographical scales. This is increasingly important due to potential impacts of climate change and woody encroachment on broad-scale landscape habitat composition, allowing the tracking of shifts in species utilisation of these changing landscapes in a way impractical using field based methods alone.

## Introduction

The manner in which large mammal herbivores interact with the plants they consume is central to the functioning of many terrestrial ecosystems (De La Dantas et al., 2016; Gordon & Prins, 2019). Plant consumption varies as a function of multiple processes including nutrient composition, phenological changes in availability, and life history traits of the consumer species. Such variation is often studied at fine scales ranging from habitat and patch quality down to bite size. However, large animals necessarily require information over large spatial scales as well (Senft et al., 1987). Furthermore, the processes that operate at, for instance, landscape levels may differ markedly from more proximate causes like plant nutrient content. This can be methodologically challenging. African savannas are characterised by still having mammalian megafaunal communities—at least in some protected areas—unlike most parts of the world where these communities are extinct (Shorrocks & Bates, 2015). Past work studying behavioural ecology of vertebrate communities often involved extensive field-based observations (Kruuk, 2003; Birkhead, Wimpenny & Montgomerie, 2014), and while this is a valuable research strategy it is time consuming and limited in the geographical extent over which these observations can be conducted. However, advances in technology allow other complimentary approaches to be applied over far larger areas, in greater detail and increasingly cost-effectively. Here, the power of such approaches is illustrated with a study combining remote sensing of vegetation and stable carbon isotope evidence designed to test the relationship between habitat and diet for the African savanna elephant (*Loxodonta africana*).

Megaherbivores in general, and elephants in particular, process large quantities of vegetation (often of poorer quality compared to the diets of smaller taxa sharing the same habitat) rather than being selective feeders (Owen-Smith, 1988). Indeed, such herbivores may play a considerable, and often underestimated, role in plant ecology (Pausas & Bond, 2019). These large animals require substantial quantities of vegetation, but in processing this bulk biomass are able to tolerate lower plant nutrient levels than most smaller herbivores (Olff, Ritchie & Prins, 2002; Müller et al., 2013; Schmitt, Ward & Shrader, 2016; Schmitt et al., 2019). This helps elephants tolerate a broad environmental range from desert to forest because they eat both browse and grass-based foods and can therefore maintain sufficient dry matter intake in a variety of habitats. Given the need to maintain very high intake rates, and their ability to eat both grass and tree products, one might expect elephant diets to reflect the availability of grass and browse in local environments. Indeed, there is evidence that more grass is eaten in open grassy environments, while browse is favoured in more wooded habitats (De Boer et al., 2000; Scholes, Bond & Eckhardt, 2003). Other studies, by contrast, did not find that elephant diets reflect the relative abundance of local vegetation (Wing & Buss, 1970; Laws, Parker & Johnstone, 1974; Codron et al., 2011), and suggest that elephants pursue strategies tending towards optimal foraging, that is those that maximise energy consumption and/or minimise exposure to potentially toxic secondary compounds (Jachmann & Bell, 1985; Stokke & Du Toit, 2000; Codron et al., 2011; Kos et al., 2012; Pretorius et al., 2012; Schmitt et al., 2019).

One potential reason for such contradictions in the studies above is that it is difficult, if not nearly impossible, to track diet and habitat at the spatial and temporal scales necessary to address the question. For instance, broad-scale habitat data, which are frequently used for such studies (Codron et al., 2011), may be too coarse to capture the scale at which elephants make foraging decisions (Shrader et al., 2012; Schmitt, Ward & Shrader, 2016; Schmitt et al., 2019). By contrast, one can get highly detailed habitat descriptions from spatially restricted sites, but it would be impractical to do this at a great many sites, especially while obtaining feeding data from each. Remote sensing, however, offers unique opportunities for mapping land-cover and quantifying vegetation characteristics over broad geographical areas at a variety of spatial, temporal and spectral scales. The increasing availability of cost-free satellite imagery means that it is now possible to conduct complementary landscape analysis linking forage availability to megafauna feeding behaviour over a large number of sites at multiple scales. Here, reanalysis of the isotope data in Codron et al. (2011) was performed using more detailed vegetation data than was available in the original study (the original study used ground-based estimates of biomass from routine ongoing monitoring programmes, which had limited spatial resolution). This investigation coupled carbon isotope analysis of faeces from the Kruger National Park (KNP), South Africa, which quickly and inexpensively provided information on proportions of browse: grass (C_3_:C_4_ plant biomass) eaten within the last 1–2 days, with remote sensing, which provided data on relative amounts of grassy vegetation at scales relevant to the daily foraging of elephants, as a way to explore links between foraging behaviour and habitat.

## Materials and Methods

### Remote sensing

African savanna vegetation often exhibits large contrasts between dry and wet seasons. Herbaceous vegetation is generally only green during the rainy season with senescence occurring shortly afterwards, whereas most woody plants remain photosynthetically active over larger parts of the year (Brandt et al., 2016). Analysis using single-date imagery alone can have limitations in discriminating between woody and herbaceous vegetation, which can be spectrally similar at certain times of year (Marston et al., 2017). To overcome this, and to quantify the varying levels of grassy vs. woody cover within the KNP study area, a pair of Landsat Enhanced Thematic Mapper (ETM+) satellite images (path 168 row 77), one acquired while herbaceous vegetation was senescent (18 June 2002) and one when grasses remained flushed (3 May 2003), were used in combination to generate a 30 m resolution land-cover map of the study area. Using a pair of images characterising vegetation state in both senescent and flushed states offered improved discrimination of woody and herbaceous vegetation based on their phenological differences. Both these image-acquisition dates fell within the period of faecal field sampling. Although the Landsat ETM+ 30 m spatial resolution precludes identification of individual trees and shrubs, Marston et al. (2017) illustrated that land-cover classifications of African savannas generated using Landsat ETM+ imagery are remarkably congruent with classifications of the same locations generated from very high resolution IKONOS and WorldView-2 imagery (which can identify individual shrubs and trees), despite some loss of spatial detail. Medium resolution Landsat ETM+ imagery is, therefore, considered appropriate for broad-scale land-cover mapping of heterogeneous African savannas.

The Landsat ETM+ surface reflectance data product, which is pre-orthorectified and atmospherically corrected, was used for this study. Further image pre-processing steps were performed using ERDAS Imagine to ensure data quality was maintained. These included: error detection and recording; cloud and cloud shadow masking; and finally compositing of the senescent and flushed vegetation images into a single dual-date composite image (Morton et al., 2011). For both images spectral bands 1 (blue, 0.45–0.52 µm wavelength), 2 (green, 0.52–0.60 µm), 3 (red, 0.63–0.69 µm), 4 (near infra-red 1, 0.77–0.90 µm), 5 (short-wave infra-red 1, 1.55–1.75 µm) and 7 (short-wave infra-red 2, 2.09–2.35 µm) were used. The composite image was projected in the Universal Transverse Mercator WGS84 zone 36 south coordinate system.

To generate training and validation data for the image classification, expert interpretation of high-resolution reference satellite imagery of the study area available via public portals such as Google Earth was used to identify locations of known land-cover types. The use of very high-resolution imagery such as that available via Google Earth as a data source for the training and validation of land-cover classifications derived from coarser resolution satellite data (such as the Landsat ETM+ imagery used here) has become an established technique (Cihlar et al., 2003; Xie, Sha & Yu, 2008; Duro, Franklin & Dube, 2012). As the acquisition dates of the Landsat ETM+ imagery and the high-resolution imagery used as a reference data source are not coincident, any areas where suspected temporal change or disturbance had occurred in the time-period between Landsat and high-resolution imagery acquisition were disregarded as a source of reference data.

For each land-cover class, the reference data points were allocated on an alternating basis as training or validation locations, which created two equal-sized datasets providing 410 locations for both the training and validation datasets (820 in total) distributed across all land-cover classes. Both training and validation datasets comprised 50 locations for each land-cover class except agriculture and closed coniferous woodland, which had 30 locations each. These two classes had lower availability of reference data due to their more restricted coverage when compared to the other land-cover classes. Although these classes were present within the Landsat image extent, they were not present within the KNP boundary which is of interest in this study. For the training data, training locations were used to generate reference polygons where spectral homogeneity allowed. Validation data was retained as point locations for classification accuracy assessment.

The classification nomenclature employed was based on a modified version of the Global Land Cover 2000 Land Cover Map of Africa classification system (Mayaux et al., 2004), classifying the forest–grassland gradient into 25% intervals (Table 1). Given the particular focus on the proportion of woody cover and grassland, the classification nomenclature followed the approach of Torello-Raventos et al. (2013), which stratified this gradient into five forest to grassland categories at 25% intervals, with the final interval at 0–5% forest. Here, the 0–5% class was amalgamated with the 5–25% class, forming a 0–25% interval class. Although not all of the land-cover classes in the classification nomenclature were used for subsequent analysis given the focus on the woodland–grassland gradient, these classes were included at the classification stage to be representative of the land-cover types found across the extent of the imagery coverage, including areas outside the KNP boundary. The land-cover classifications were performed using a random forest classifier in R, using the random Forest package (Liaw & Wiener, 2002). Classification accuracy assessment was subsequently performed using the validation dataset.

**Table 1:** Land cover map classification nomenclature.

General habitat	Land cover class and code
Woodland	Closed deciduous woodland (CDW) (75–100% woody cover)
	Open deciduous woodland (ODW) (50–75% woody cover)
Grassland	Discontinuous grassland (DG) (25–50% woody cover) Continuous grassland (CG) (0–25% woody cover)
Anthropogenic classes	Agriculture (AG)
	Built-up (BU)
	Closed coniferous woodland (CCW) (non-indigenous forestry plantations)
Bare	Bare ground (BA)
Water	Water (W)

Land-cover data were extracted for a series of concentric circular buffers surrounding each isotope sample site (see below) of radius sizes 2, 4, 8 and 12 km. The proportion of each land-cover class was calculated using the ArcMap 10.2 and Geospatial Modelling Environment software packages.

### Stable isotopes

**Figure 1:**
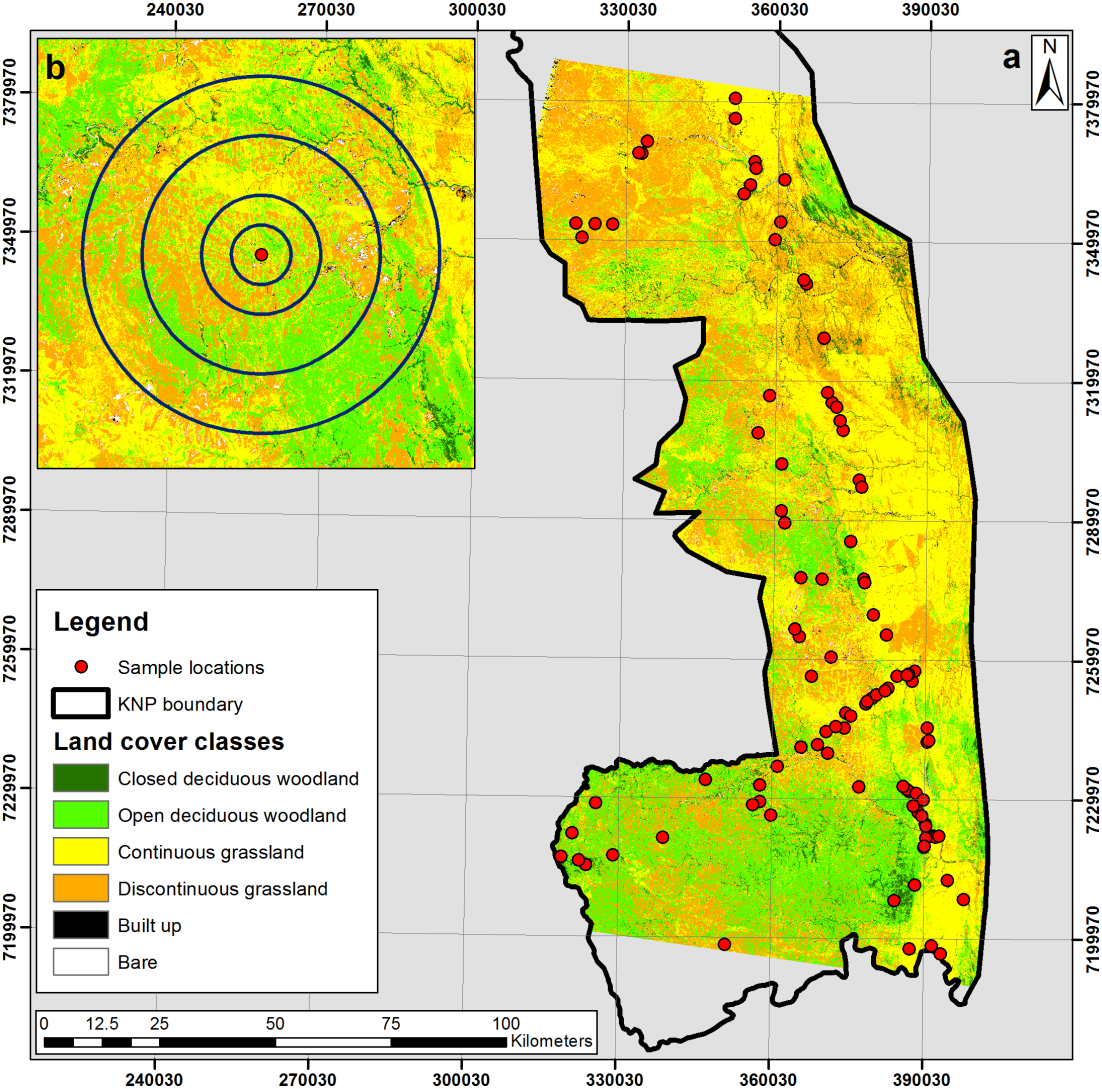
(A) Land cover classification of the southern Kruger National Park study area; and (B) inset image displaying 2 km, 4 km, 8 km and 12 km concentric buffers surrounding an isotope sample location.

Faecal isotope data for elephants are derived from specimens collected from south of the Olifants River in the KNP, South Africa, from June 2002 until January 2005–a period of average rainfall, falling outside climatically extreme periods of severe drought (1991–1992 and 2014–2016), or high rainfall years (1999–2000) in the park (MacFadyen et al., 2018; Staver, Wigley-Coetsee & Botha, 2019). Faecal sampling protocols were permitted by South African National Parks and carried out in accordance with their guidelines for fieldwork. Specimens were obtained across four sub-regions representing a variety of savanna landscape types across the southern portion of Kruger Park (Fig. 1), and include collections made at monthly and at seasonal (biannual) resolutions (Codron et al., 2011). For the first 2 years, collections were made biannually, once in the dry season and once in the wet. Thereafter, sampling was carried out at monthly intervals, with the aim of collecting at least 10 individual faecal specimens per study region per month. Only recent faeces (i.e. fresh or damp) were collected. Each specimen encountered was assumed to represent a separate individual, a reasonable assumption because no more than a single sub-region was sampled per day. Further, because only fresh material was collected, it is unlikely that sampling of multiple individuals per day was a frequent occurrence. However, because sampling was opportunistic, the demographic status of individuals could not be ascertained.

Faeces were oven-dried at 60 °C for 24 h and then ground through a one mm sieve to a homogeneous powder. Samples were combusted individually in an automated elemental analyser (Carlo–Erba, Milan, Italy), and the resultant CO_2_ gas introduced to a mass spectrometer (MAT 252 or DELTA XP; Finnigan, Bremen, Germany) using a continuous flow-through inlet system. ^13^C/^12^C ratios were presented in delta (δ) notation, in per mill (‰) relative to the VPDB (Venice Pee Dee Belemnite) standard (Table S1). Standard deviations of repeated measurements of internal and interlaboratory plant and protein standards were less than 0.1%. The results were converted to estimates of %C_4_ food intake using a linear mixing model that accounted for predicted diet-faeces isotopic discrimination effects and known spatial and temporal changes in plant carbon isotope compositions (see Codron et al., 2011 for full methodology).

### Range sizes

Elephants feed throughout the day, with gut retention time in captive African elephants recorded as less than 30 h (Hackenberger, 1987; Clauss et al., 2007). Thomas, Holland & Minot (2012) presented several years of GPS data collected on three female elephants in the southern KNP, which gave a mean minimum daily distance moved of about 4 km. These values were used to analyse the land-cover within a series of concentric buffers related to the likely range from which the faecal samples were derived over the course of a day (4 km) as well as smaller (2 km) and larger (8 and 12 km) range sizes (Fig. 1). The land-cover data were then correlated with the stable isotope results with permutation tests performed to determine whether or not there is any relationship between the proportion of C_4_ in the environment and the proportion of C_4_ in the diet.

### Grassiness index

As the Landsat imagery is medium resolution (30 × 30 m per pixel) it is not possible to discriminate individual trees. Therefore, the land-cover classification calculated the proportion of woody cover within each pixel on a 4-point scale: closed deciduous woodland (>75% woody cover), open deciduous woodland (50–75% woody cover), discontinuous grassland (25–50% woody cover) and continuous grassland (<25% woody cover). A ‘grassiness index’ was calculated by multiplying the percentage of relevant land-covers per buffer by a modifier (from ×1 for closed woodland to ×4 for open grassland) and then summing them together. Therefore for a given percentage of pixels in a buffer ‘continuous grassland’ would contribute four times more to the index than would closed deciduous woodland; so buffers with higher values contain more grass than those with lower values. Of the many potential indices that could be devised this one has the virtue of simplicity (Murtaugh, 2007), and importantly was decided on in advance of any quantitative analyses to avoid the danger of trying multiple indices before settling on the one with the most interesting results (Amrhein, Trafimow & Greenland, 2019). Once calculated, the grassiness index for each sample and buffer size was plotted against the %C_4_ from the faecal samples.

The hypothesis that elephant %C_4_ intake is related to grassiness in the landscape was tested using simple linear regressions of estimated %C_4_ in the diet on the grassiness index for each sampling locality and time interval (month). However, this analysis may not be robust to bias introduced by the fact that most localities (128 of 194) are represented by more than one faecal specimen (in fact, more than a third of localities were presented by four or more faecal specimens, in any given sampling interval). Therefore, permutations tests were used, by randomising the data for each locality and sampling interval, and summarising the regression results over 10^3^ iterations. This allowed us to compute permuted means and 95% confidence limits for the regression parameters *r*^2^, the intercept (*a*), and slope (*b*) that were unbiased with respect to repeated collections at a given locality. Significance tests were based on Monte Carlo analysis of resultant model *p*-values, where (with α-level set at 0.05):

$$\hat p = 1 - \displaystyle{{{\rm sum\; of\; models\; with\; }p < 0.05} \over {{{10}^3}}}$$

Models were repeated for all the data combined, as well as for wet and dry season data separately. A similar Monte Carlo approach was used to compare *r*^2^ and *b* of the two seasons, but in this case the resultant *p*-values were divided by two for a one-tailed test, with the null hypothesis: dry ≤ wet season. These procedures were repeated for grassiness values obtained from each of the four buffer sizes for comparison. Regression models and the permutation algorithm were carried out using R v 3.5.2 (R Core Team, 2015).

## Results

The land-cover classification generated, clipped to the area of the KNP, is displayed in Fig. 1. The land-cover class coverages within this clipped area comprised 40.69% continuous grassland, 32.58% discontinuous grassland, 21.56% open deciduous woodland, 4.10% closed deciduous woodland, 0.57% bare ground, 0.36% built-up and 0.13% water. The overall accuracy of the classification was 90.73%, with a detailed confusion matrix presented in Table 2.

**Table 2:** Land cover classification confusion matrix. The land cover classification is compared to validation locations of known land cover type (*n* = 410), with overall classification accuracy high at 90.73%. For the key woodland and grassland classes of interest, class-specific accuracies were generally high with closed deciduous woodland, open deciduous woodland and continuous grassland all displaying producer’s accuracies at or over 90%, and user’s accuracies over 83%. Discontinuous grassland had lower accuracies of 76% and 77.55% for producer’s and user’s accuracies respectively. Land cover class abbreviations: CDW, closed deciduous woodland; ODW, open deciduous woodland; CG, continuous grassland; DG, discontinuous grassland; AG, agriculture; BU, built-up; CCW, closed coniferous woodland; BA, bare; and W, water. UA, Users Accuracy; PA, Producers accuracy.

Classified data	Reference data									
	CDW	ODW	CG	DG	AG	BU	CCW	BA	W	UA (%)
CDW	49	3	0	2	3	0	0	0	0	85.96
ODW	0	45	0	5	2	0	2	0	0	83.33
CG	0	0	48	5	0	2	0	0	0	87.27
DG	0	2	2	38	0	7	0	0	0	77.55
AG	1	0	0	0	25	0	0	0	0	96.15
BU	0	0	0	0	0	40	0	1	0	97.56
CCW	0	0	0	0	0	0	28	0	0	100.00
BA	0	0	0	0	0	0	0	49	0	100.00
W	0	0	0	0	0	1	0	0	50	98.04
PA (%)	98.00	90.00	96.00	76.00	83.33	80.00	93.33	98.00	100.00	
Overall accuracy (%) = 90.73										

**Figure 2:**
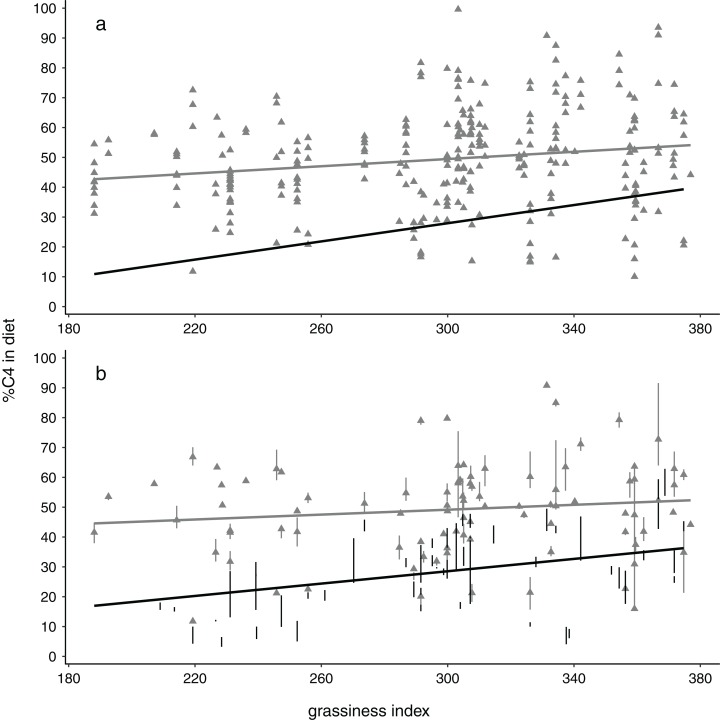
Relationships between %C_4_ grass in elephant diets with availability of grass in the landscape, depicted showing (A) all data points and (B) means and interquartile ranges for each collection locality. Note the steeper slopes and stronger relationships for dry season data (black circles, black regression lines) compared with wet season data (grey triangles, grey lines). All results shown here are for the 4 km buffer size.

Monte Carlo one-tailed permutation tests showed relationships between %C_4_ intake and grassiness to be positive regardless of season (Fig. 2). However, models only met the conventional significance level (*p* < 0.05) when using data for dry seasons or, usually, both seasons combined (Table 3). Wet-season models were non-significant and concomitantly had substantially smaller slopes and *r*^2^ values than models for the dry season. Interestingly, the slopes of these models tended to increase with increases in buffer size, at least in the case of dry season data (non-overlapping confidence estimates in Table 3). This implies that, in terms of the predicted relationship between diet and habitat structure, the effect becomes increasingly apparent over larger spatial scales. Note that the size of the effects (slope and *r*^2^) are likely much more biologically meaningful than the *p*-values (significance values) (Nakagawa & Cuthill, 2007).

**Table 3:** Permutation test permuted means and 95% confidence limits for the regression parameters *r*^2^, intercept, and slope (*b*) and significance test *p*-values. *n* = number of observations per location; regression parameters are shown as means with 95% confidence intervals in parentheses; *p*-values are derived from Monte Carlo simulations of 10^3^ randomised permutations, adjusted to one-tailed equivalents for the last two columns; significant models are shown in bold.

						Significance tests (*p*-values)		
Buffer size (km)	Season	*n*	*r*^2^	Intercept	Slope (*b*)	model	*r*^2^_dry > wet_	*b*_dry > wet_
2	Both	194	**0.0359 (0.0353–0.0364)**	**17.0890 (16.9196–17.2584)**	**0.0710 (0.0704–0.0716)**	**0.022**		
2	Dry	101	0.0613 (0.0602–0.0624)	8.0449 (7.8404–8.2494)	0.0687 (0.0681–0.0694)	0.086		
2	Wet	93	0.0172 (0.0165–0.0179)	36.7310 (36.4762–36.9858)	0.0413 (0.0404–0.0422)	0.962	<0.0001	<0.0001
4	Both	194	**0.0424 (0.0418–0.0430)**	**13.7514 (13.5721–13.9308)**	**0.0819 (0.0813–0.0826)**	**<0.01**		
4	Dry	101	**0.1202 (0.1187–0.1218)**	**−2.3335 (−2.5697–2.0973)**	**0.1029 (0.1021–0.1037)**	**<0.0001**		
4	Wet	93	0.0160 (0.0153–0.0167)	36.7965 (36.5121–37.0810)	0.0412 (0.0402–0.0422)	0.969	<0.01	0.036
8	Both	194	**0.0373 (0.0367–0.0379)**	**12.1572 (11.9360–12.3785)**	**0.0881 (0.0873–0.0889)**	**0.026**		
8	Dry	101	**0.1349 (0.1333–0.1365)**	**−9.3957 (−9.6741–9.1174)**	**0.1271 (0.1262–0.1281)**	**<0.0001**		
8	Wet	93	0.0141 (0.0135–0.0148)	36.3013 (35.9753–36.6273)	0.0434 (0.0422–0.0446)	0.988	<0.0001	<0.01
12	Both	194	0.0257 (0.0252–0.0261)	14.9687 (14.7473–15.1901)	0.0803 (0.0795–0.0811)	0.237		
12	Dry	101	**0.1189 (0.1176–0.1203)**	**−10.9163 (−11.1901–10.6424)**	**0.1341 (0.1331–0.1350)**	**<0.0001**		
12	Wet	93	0.0235 (0.0227–0.0244)	30.8930 (30.5453–31.2408)	0.0634 (0.0621–0.0647)	0.890	<0.0001	<0.0001

A possible factor in the analysis is the presence of water (such as boreholes or waterholes) within the buffer. These are more likely to have more grass surrounding them, contrasting with rivers which are more likely to have riparian woodlands.

## Discussion

These results are consistent with elephant diets tracking the proportion of grass in the habitat, but only during periods where grass quality and/or availability is low, that is during the dry season. This potentially harmonises seemingly contrasting suggestions that elephants select vegetation in proportion to its abundance (De Boer et al., 2000; Scholes, Bond & Eckhardt, 2003; Owen-Smith & Chafota, 2012; Schmitt, Ward & Shrader, 2016; Schmitt et al., 2019) with reports that they selectively consume grass (Guy, 1976; Owen-Smith, 1988). It appears that both assertions are true in the KNP depending on season. On balance, however, it may be best to categorise these elephants as selective feeders that are capable of maintaining broader diets when dealing with some form of constraint–in this case the low quality and/or abundance of grass in the dry season. This is also broadly consistent with other reports that elephants are selective feeders that eat large quantities of grass in the wet season and switch to woody plant species, and a variety of low-quality plant parts, in the dry season (Barnes, 1982; Owen-Smith & Chafota, 2012). More proximate factors such as soil geochemistry (and its effects on plant nutrient content) and avoidance of secondary metabolites in woody vegetation (Schmitt, Ward & Shrader, 2016; Schmitt et al., 2019) may also play a role in food selection, although it is unclear that such fine-scale foraging decisions occur over landscape scales—the scale of our study. Moreover, we observed that the influence of grass availability on grass intake became increasingly apparent over broader spatial scales, which could imply that decisions about whether to eat browse or grass are constrained only by relative availability, whereas finer-scale decisions determine which plant species or organs are consumed.

The idea that animals may forage in an optimal manner—or at least tend towards optimal foraging—dates back to the mid 1960’s but was developed in more theoretical detail during the 1970’s (Krebs & McCleery, 1984). In this context, the tendency towards higher grass consumption with increased availability might be expected, at least across woodland to grassland environments, as grasses tend to be fairly continuously distributed across such landscapes (hence available in bulk), are probably more consistent in nutritional value (i.e. they have no indigestible woody parts), and probably allow higher harvesting rates. These qualities outweigh, or at least match, potential losses of grazing given the low nutrient content of grasses (Hummel et al., 2006). Because grasses can be consumed without the woody stems that often accompany elephant tree-leaf consumption (Owen-Smith & Chafota, 2012), digestible dry-matter intake should typically be higher when elephants consume grasses. The non-preference for grasses during the dry season implies that nutritional and harvesting benefits for grass relative to browse becomes neutral during this time, resulting in diets which are largely a function of relative availability of the resource, that is encounter rate. Thus, one might predict, for instance, that in areas where grass stays green for the greater part of the year due to close proximity to permanent water, there may be no relationship between diet and habitat even seasonally. A strong preference for grasses makes sense given that the elephant dental morphology appears to be an adaptation for grass consumption (Lister, 2013), and that carbon isotope evidence from the enamel of post-Miocene fossil elephantids from Africa shows a strong preference for C_4_ grasses (Cerling, Harris & Leakey, 1999). In this light, the high levels of browse consumption by many modern elephant populations seem puzzling (for example, in our study C_3_ browsing is a major part of the diet—Fig. 2). Evidently, elephants readily bulk up their diets with grasses when this resource is available, seemingly favouring this feeding style when resources are not limiting, as in our rainy season data.

Nonetheless, it is worth noting that our results are somewhat different from those presented in Codron et al. (2011) which, using the same carbon isotope dataset, found no evidence that elephant browse and grass consumption tracked local availability. One possible reason for this is that the current study focused on the southern portion of KNP, whereas the former included the northern region in which the tree communities are dominated by a single-species: mopane *Colophospermum mopane*. Given the high concentration of defensive secondary compounds in mopane (Styles & Skinner, 2000; Kohi et al., 2010), and the tendency of many (but certainly not all) animals to avoid high loads of such anti-feedants by diversifying their diets (Freeland & Janzen, 1974; Wiggins, McArthur & Davies, 2006; Torregrossa, Azzara & Dearing, 2011; Wilkinson & Sherratt, 2016), elephants might under-utilize mopane. Such a foraging behaviour would necessitate greater consumption of grasses in habitats such as the northern KNP, and consequently reduce any relationship between browse/graze consumption and availability for the park as a whole. Indeed, Codron et al. (2011) suggested a limited effect of mopane on increasing grass consumption—the effect size in this case was probably reduced by including data from southern KNP. Kos et al. (2012), however, found no evidence that elephants restricted their use of mopane in a study in a reserve adjacent to KNP–speculating that the extent to which elephants avoid this tree may be affected by the availability of other plants in the area. One possibility is that elephants may in some situations need to include other plant species to access micro-nutrients which may be scarce in the dominant plant species (Westoby, 1978).

Another reason for the difference between the current study and that of Codron et al. (2011) is that for the latter, vegetation-cover data were based on calculated averages across large areas, so may not have reflected the actual areas in which the elephants were feeding and depositing the faecal samples, while the present study used habitat data at a much finer resolution. This illustrates the importance of coupling isotope and remote-sensing data. Carrying out ground-based vegetation surveys for the more than 1,000 faecal samples from 149 collection sites in Codron et al. (2011) would have been extremely impractical, time intensive and expensive. Here, by contrast, once the satellite imagery had been classified and the models were validated, extracting grassiness index data from the land-cover classification for each site was comparatively trivial. It is, however, acknowledged that further work is also needed to investigate how variations in ranging patterns may influence observed results.

The increasing availability of cost-free remote sensing imagery, along with the improving image spatial resolution and temporal repeat coverage, means that future work could enable the characterisation of land-cover, and therefore food resources (using the methods presented here), at multiple dates throughout the year where cloud-free imagery allows. Therefore, seasonal changes in the relative C_3_ and C_4_ availability within a landscape can potentially be quantified, and in turn related to isotopic values derived from faecal sampling conducted in different seasons. Along with the ability to perform multi-scale examination of the landscape around faecal sampling points using different buffer sizes, this creates a highly flexible tool which, complementary to existing methods, offers an improved ability to monitor and predict animal behavioural patterns at broad geographical scales. This is increasingly important due to the potential impacts of climate and other environmental changes such as woody encroachment on broad-scale landscape habitat composition (Warren et al., 2001; Huntley et al., 2016), as the use of isotope data in combination with remote-sensing analysis will be able to track shifts in species utilisation of these changing landscapes. These methods are also transferable both to other geographical areas, and to other species. The ability to apply multiple scales of observation guided by established species-specific home ranges is also useful where there is a potential disconnect between where animals are feeding and defaecating. For example, in our study the massive size and large daily ranging pattern of elephants creates a potential disconnect between foraging and defaecation sites which could lead to ambiguity in linking diet with habitat. Despite this, significant correlations were found between grassiness and C_4_ intake at all buffer sizes, with the strongest correlations observed for the 4 and 8 km buffer sizes. We would argue that this result is robust given that it persists across buffer sizes. This result suggests that the uncertain relationship between where the elephants ate and where they defaecated does not present a confounding factor at least for this species and/or at this spatial scale of sampling. It can thus be predicted that our approach would yield even more accurate results for smaller-bodied species that travel over shorter distances than elephants.

## Conclusions

Our results suggest that one reason for the sometimes contradictory results in studies of feeding selectivity in elephants is that there is variation in preference throughout the year. In our study, elephants consumed C_4_ vegetation in proportion to its availability in the surrounding area during the dry season, but during the rainy season there was less of a relationship between C_4_ intake and availability as elephants targeted grasses in these periods. Over the last few decades various technological advances (for example, GPS collars, camera traps, DNA technology) have greatly added to the ease of collecting some types of data in behavioural ecology and conservation biology (Schaller, 2012). Here, we demonstrated the utility of comparing isotopes and remote sensing. Clearly, any study utilising isotopes is considering diet in a very coarse way compared to direct observational studies or models which make use of data on plant consumption at the species level (Schmitt, Ward & Shrader, 2016), which can focus on both species and plant part consumed. Studies such as the above will never supplant these traditional studies given the rich detail they provide, and the very different questions they can address. However, the importance of the approach outlined in this paper is that it allows us to address diets of wildlife species over a very large and vegetationally heterogeneous area—greatly increasing the data available to inform management in the face of an often rapidly changing world. The potential of this approach is shown by the way it potentially explains contradictory results in previous studies of elephant foraging.

## Supplemental Information

10.7717/peerj.8622/supp-1 Supplemental Information 1 Stable carbon isotope and remote-sensing derived land cover class coverage raw data for sample locations across the southern Kruger National Park, South Africa.

## Additional Information and Declarations

### Data Availability

The following information was supplied regarding data availability: The raw data are available as a Supplemental File. This includes stable carbon isotope data, sampling locations and proportional land cover class coverage within 2, 4, 8 and 12 km buffered areas centred on each sampling location respectively.
